# Effects of bone turnover status on the efficacy and safety of denosumab among haemodialysis patients

**DOI:** 10.1038/s41598-022-12029-3

**Published:** 2022-05-11

**Authors:** Mayuko Hori, Kaoru Yasuda, Hiroshi Takahashi, Chika Kondo, Yuichi Shirasawa, Yuka Ishimaru, Yuka Sekiya, Kunio Morozumi, Shoichi Maruyama

**Affiliations:** 1 Department of Nephrology, Masuko Memorial Hospital, 35-28 Takehashi-cho, Nakamura-ku, Nagoya, Aichi 453-8566 Japan; 2 Department of Nephrology, Fujita Health University School of Medicine, Toyoake, Aichi Japan; 3 Department of Nephrology, Nagoya University Graduate School of Medicine, Showa-ku, Nagoya, Aichi Japan

**Keywords:** Biomarkers, Medical research, Nephrology

## Abstract

Denosumab is reported to increase bone mineral density (BMD) among haemodialysis patients; however, hypocalcaemia is a serious adverse effect among chronic kidney disease (CKD) patients. Identifying which patients will show greater improvement in BMD is important. We enrolled 84 haemodialysis patients with osteoporosis in our study. 28 patients initiated denosumab treatment between October 2019 and October 2020. We assessed BMD changes and investigated the association between baseline bone turnover marker (BTM) levels and 6-month changes in BMD after denosumab treatment. BMD was increased at 6 months in denosumab-treated patients compared with patients not treated with denosumab (lumbar spine: 5.34% vs. − 0.49%; total hip: 2.43% vs. − 0.47%). Bone-specific alkaline phosphatase (BAP) and tartrate-resistant acid phosphatase-5b (TRACP-5b) at baseline were independently associated with increased BMD in the total hip (BAP: β = 0.472, p value = 0.004; TRACP-5b: β = 0.433, p value = 0.008) and lumbar spine (BAP: β = 0.591, p value = 0.001; TRACP-5b: β = 0.613, p value = 0.0008). BAP and TRACP-5b were also independent predictors of hypocalcaemic events (OR [95% CI] 1.747 [1.084–4.604] and 1.006 [1.000–1.015], respectively). BTMs may be associated with increased BMD and hypocalcaemic events after denosumab treatment. BTM measurement may be useful for assessing the effect of denosumab on BMD; however, careful monitoring of serum calcium levels is needed.

## Introduction

Low bone mass and fracture are more common complications among end-stage renal disease (ESRD) patients than in the general population, and they have high morbidity and mortality^1,2^. Management for osteoporosis among haemodialysis (HD) patients has been an important topic because the choices of treatment have increased with advances in medicine. In the general population, treatment with bisphosphonates has been established for fracture prevention. On the other hand, the long-term safety of bisphosphonates has been controversial in HD patients because these compounds are excreted via the kidney^3^.

Denosumab is a fully human monoclonal antibody against receptor activator of nuclear factor-kappa-Β ligand (RANKL), which is an essential cytokine for osteoclast function^4,5^. Denosumab decreases bone resorption and increases bone mineral density by decreasing the number and activity of osteoclasts^6^. Because denosumab is not eliminated by the kidney, this drug is thought to be better suited for patients with chronic kidney disease (CKD)^7^. Some past studies have shown the effect of denosumab to reduce the risk of fracture among patients with advanced CKD^8,9^. The effect of denosumab in restoring bone mass among HD patients has also been reported^10,11^. Conversely, several clinical studies of ESRD patients showed that hypocalcaemia, the result of “hungry bone-like syndrome”, was a common adverse event after initiating treatment with denosumab in HD patients^12^.

Recent studies have shown that a higher tartrate-resistant acid phosphatase-5b (TRACP-5b) level, which reflects high bone turnover, is a predictor of hypocalcaemic events^13,14^. However, little is known about markers for predicting the effect of denosumab. When considering initiating treatment for osteoporosis in HD patients, a marker to help identify the patients in whom denosumab would have a good effect on BMD would be useful.

The aim of this study was to investigate the factors that increase BMD after initiating treatment with denosumab.

## Results

### Patient characteristics

We enrolled 95 haemodialysis patients who were diagnosed with osteoporosis according to the Japanese Society for Bone and Mineral Research criteria. Among them, 29 patients chose to begin treatment with denosumab, and denosumab treatment was initiated between October 2019 and October 2020. Among the patients treated with denosumab, 1 patient died of pneumonia before the 6-month bone mineral density (BMD) measurement and was excluded from this analysis. Among the 66 control patients, 10 patients were excluded because they did not undergo BMD measurements at 6 months (death: 3 patients; changed hospitals: 1 patient; other reasons: 6 patients).

Their clinical data (denosumab group: 28 patients; control group: 56 patients) at baseline are shown in Table 1. No significant differences were found in patient demographics, serum phosphate, serum magnesium, serum albumin, serum intact parathyroid hormone (PTH), total hip BMD, past history or medication between the two groups.

**Table 1:** Baseline characteristics in the denosumab and control groups.

	Denosumab n = 28	Control n = 56	P value
**Demographics**			
Age (year)	67.6 ± 11.2	71.4 ± 13.4	0.18
Sex (%), male	46.4	64.3	0.11
BMI (kg/m^2^)	19.7 ± 2.3	20.6 ± 2.7	0.78
DM (%)	35.7	37.5	0.87
HD vintage (year)	7.6 (4.3, 16.7)	8.6 (3.8, 13.6)	0.83
HD duration (h/week)	12 (12, 13.5)	12 (12, 12)	0.50
**Laboratory data**			
BUN (mg/dL)	60.9 ± 13.2	57.1 ± 14.9	0.22
Adj. calcium (mg/dL)	9.5 ± 0.4	9.0 ± 0.5	0.0002
Phosphate (mg/dL)	4.9 ± 0.9	5.0 ± 1.2	0.88
Magnesium (mg/dL)	2.5 ± 0.3	2.4 ± 0.3	0.12
Alb (g/dL)	3.6 ± 0.3	3.4 ± 0.3	0.057
Hb (g/dL)	11.5 ± 0.8	11.4 ± 1.1	0.47
ALP (IU/L)	259 (201, 341.3)	242 (195.5, 304.5)	0.53
iPTH (pg/mL)	132.5 (72.3, 178.3)	142.5 (83.7, 178)	0.60
BAP (µg/L)	12.7 (10.5, 25.0)		
TRACP-5b (mU/dL)	490.5 (255.7, 659.2)		
**Bone mineral density**			
Total hip BMD, g/cm^2^	0.593 (0.555, 0.655)	0.636 (0.551, 0.696)	0.21
Lumbar spine BMD, g/cm^2^	0.860 (0.765, 1.008)	0.955 (0.874, 1.069)	0.015
**Past history**			
CVD (%)	32.1	33.9	0.86
Parathyroidectomy (%)	0.0	3.5	0.19
Incident fracture (%)	39.3	37.5	0.87
**Medication**			
CaCO_3_ (%)	39.3	35.7	0.74
Phosphate binders (%)	71.4	64.2	0.50
Vitamin D (%)	96.4	87.5	0.15
Alfacalcidol (%)	10.7	28.6	
Calcitriol (%)	35.7	8.9	
Eldecalcitol (%)	0.0	1.8	
Maxacalcitol (%)	50.0	48.2	
Calcimimetics (%)	35.7	50.0	0.21
PPI (%)	53.6	64.3	0.34
Bisphosphonate (Before denosumab)	10.7		

Data are expressed as the means ± SDs or medians (interquartile ranges).

*BMI* body mass index, *DM* diabetes mellitus, *BUN* blood urea nitrogen, *Adj* calcium, albumin-adjusted calcium, *Alb* albumin, *Hb* haemoglobin, *ALP* alkaline phosphatase, *iPTH* intact parathyroid hormone, *BAP* bone-specific alkaline phosphatase, *TRACP-5b* tartrate-resistant acid phosphatase-5b, *CVD* cardiovascular disease, *CaCO*_*3*_ calcium carbonate, *PPI* proton pump inhibitors.

Significant differences were found in serum albumin-adjusted calcium (Adj Ca) and L2-L4 BMD.

### Change in BMD

**Figure 1:**
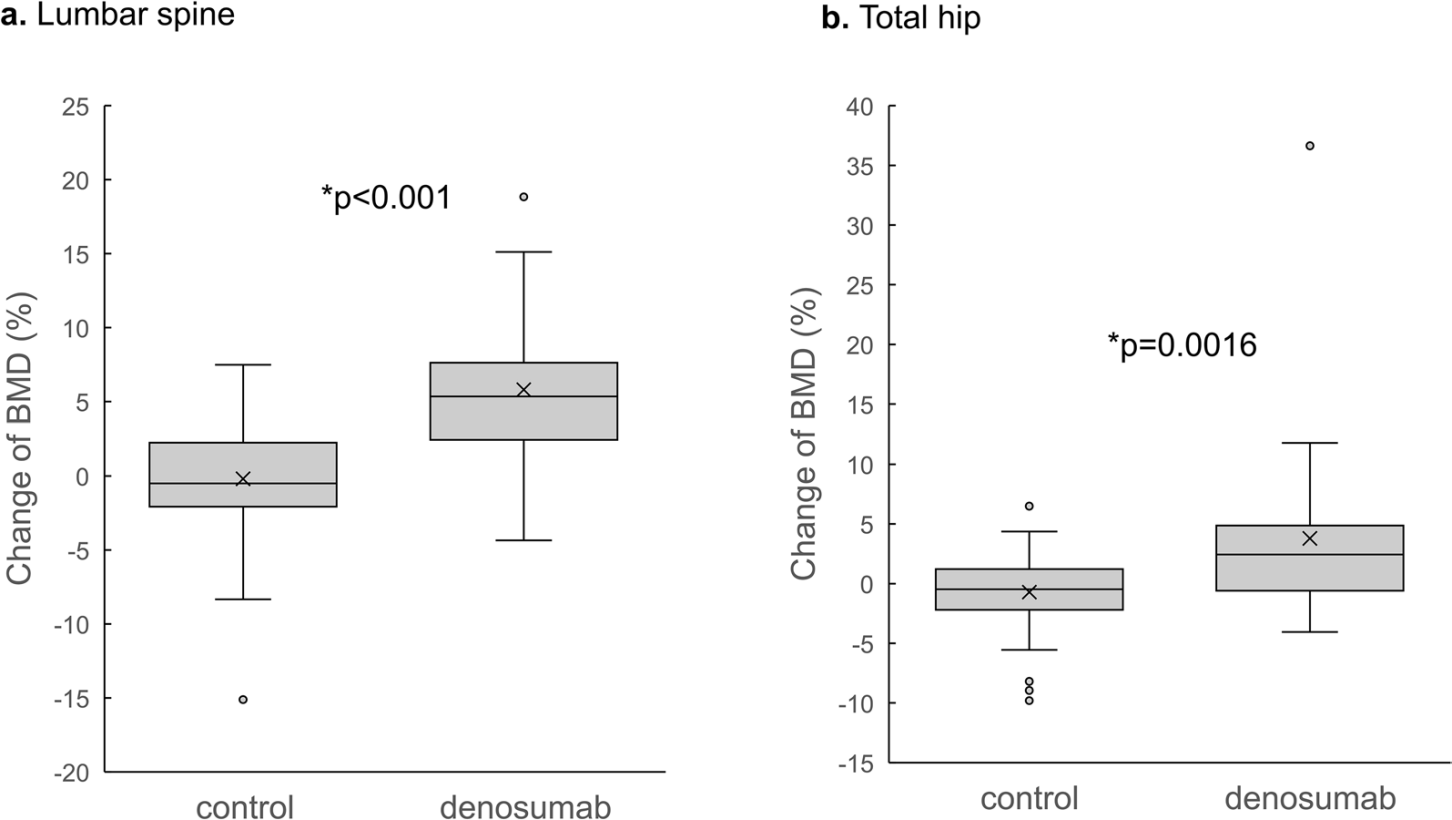
Median change in BMD (%) in the lumbar spine (**a**) and total hip (**b**) at 6 months from baseline. Error bars, IQR; *, significantly different from ANCOVA adjusted for serum adjusted calcium levels and L2-L4 BMD.

Figure 1 shows the change in BMD % as the medians and interquartile ranges (IQRs) in the lumbar spine and total hip in the two groups from baseline to 6 months. When we used analysis of covariance (ANCOVA) with the covariates that were different between the two groups, the changes in BMD %, median (IQR) from baseline in the denosumab group were 5.34 (2.44, 7.62) % in the lumbar spine and 2.43 (− 0.58, 4.86) % in the total hip, which were significantly higher than those in the control group [− 0.49 (− 2.07, 2.25) % in the lumbar spine; − 0.47 (− 2.19, 1.21) % in the total hip]. Hence, in the denosumab group, some patients had BMD decreases at 6 months (lumbar spine: 5 patients; total hip: 7 patients). A patient had more than 35% of BMD change-rate in total hip. In this case, the BMD change-rate was calculated as high due to the extremely low baseline BMD.

### The effect of bone turnover markers on 6-month BMD changes

**Figure 2:**
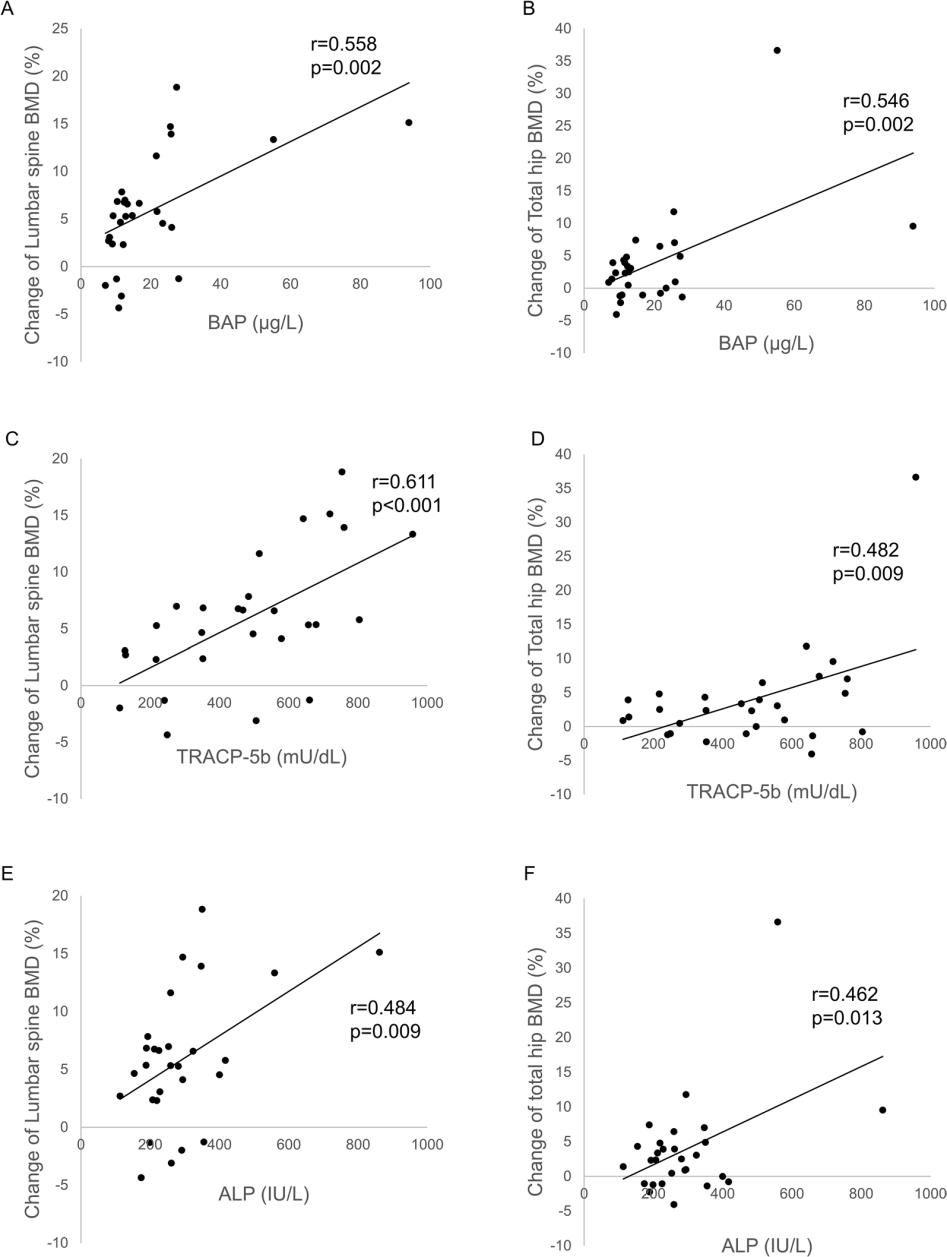
Correlation between BTMs at baseline and change in BMD. (**a**) BAP and change in BMD in lumbar spine, (**b**) BAP and change in BMD in total hip, (**c**) TRACP-5b and change in BMD in lumbar spine and (**d**) TRACP-5b and change in BMD in total hip, (**e**) ALP and change in BMD in lumbar spine and (**f**) ALP and change in BMD in total hip.

We investigated the association between bone turnover markers (BTMs) and changes in BMD in the lumbar spine and total hip after initiation of denosumab. BTMs at baseline showed positive correlation with change in BMD in the lumbar spine (BAP: r = 0.558, p = 0.002; TRACP-5b: r = 0.611, p < 0.001; ALP: r = 0.484, p = 0.009) and total hip (BAP: r = 0.546, p = 0.002; TRACP-5b: r = 0.482, p = 0.009; ALP: r = 0.462, p = 0.013) (Fig. 2). Simple regression analyses showed that bone-specific alkaline phosphatase (BAP), TRACP-5b and alkaline phosphatase (ALP) at baseline may be significant predictors of 6-month BMD changes in both the lumbar spine (BAP: β = 0.558, p = 0.002; TRACP-5b: β = 0.611, p = 0.0005; ALP: β = 0.484, p = 0.009) and total hip (BAP: β = 0.546, p = 0.002; TRACP-5b: β = 0.482, p = 0.009; ALP: β = 0.462, p = 0.013) (Table 2). Adjustment for age and sex using multiple regression analyses did not change the significant association between BTMs and 6-month BMD changes in both the lumbar spine (BAP: β = 0.591, p = 0.001; TRACP-5b: β = 0.613, p = 0.0008; ALP: β = 0.512, p = 0.007) and total hip (BAP: β = 0.472, p = 0.004; TRACP-5b: β = 0433, p = 0.008; ALP: β = 0.423, p = 0.011) (Table 3).

**Table 2:** Factors affecting BMD changes in the total hip and lumbar spine at 6 months after treatment with denosumab: simple regression analyses.

Variables	Total hip		Lumbar spine	
	β	p value	β	p value
Age (year)	− 0.412	0.029	− 0.120	0.54
Sex, male	− 0.269	0.16	0.096	0.62
BMI (kg/m^2^)	− 0.314	0.10	− 0.226	0.24
DM	− 0.278	0.15	− 0.204	0.29
HD vintage (year)	0.331	0.084	0.245	0.20
HD duration (h/week)	0.011	0.95	− 0.100	0.61
BUN, mg/dL	− 0.041	0.83	− 0.271	0.16
Adj. Ca, mg/dL	0.027	0.88	− 0.144	0.46
P, mg/dL	− 0.157	0.42	− 0.303	0.11
Mg, mg/dL	− 0.061	0.75	− 0.223	0.25
Alb, g/dL	− 0.021	0.91	− 0.193	0.32
Hb, g/dL	− 0.255	0.18	-0.190	0.33
ALP, IU/L	0.462	0.013	0.484	0.009
iPTH, pg/mL	0.155	0.43	0.173	0.37
Past history of CVD	0.225	0.24	− 0.278	0.15
Past incident fracture	− 0.068	0.72	0.122	0.53
CaCO_3_	0.298	0.12	− 0.202	0.30
Other phosphate binders	0.0053	0.97	0.091	0.64
Vitamin D	0.074	0.70	0.057	0.77
Calcimimetics	− 0.170	0.38	0.0047	0.98
PPI	0.015	0.93	− 0.264	0.17
BAP, μg/L	0.546	0.002	0.558	0.002
TRACP-5b, mU/dL	0.482	0.009	0.611	0.0005
25 (OH) vitamin D	− 0.260	0.18	− 0.304	0.11
1,25 (OH) 2 vitamin D3	− 0.136	0.48	− 0.0089	0.96

*β* standardized regression coefficient, *BAP* bone-specific alkaline phosphatase, *TRACP-5b* tartrate-resistant acid phosphatase-5b.

**Table 3:** The effect of bone turnover markers on the change in BMD in the total hip and lumbar spine at 6 months: adjusted for sex and age.

Variables	Total hip		Lumbar spine	
	β	p value	β	p value
ALP, IU/L	0.423	0.011	0.512	0.007
BAP, μg/L	0.472	0.004	0.591	0.001
TRACP-5b, mU/dL	0.433	0.008	0.613	0.0008

*β* standardized regression coefficient, *ALP* alkaline phosphatase, *BAP* bone-specific alkaline phosphatase, *TRACP-5b* tartrate-resistant acid phosphatase-5b.

### Adverse effects

Although none of our patients experienced allergies or jaw osteonecrosis, 16 patients (57.1%) experienced hypocalcaemia with Adj Ca < 8.0 mg/dL. Only 1 patient had a temporary sense of fatigue, and the others had asymptomatic hypocalcaemia. Among the patients, 12 patients had nadir Ca levels after the 1st administration; 1 patient, after the 2nd administration; and 3 patients, after the 3rd administration. 23 patients (82.1%) experienced a large decline in serum Ca, defined as a maximum calcium decline ≥ 1.5 mg/dL or ≥ 1.0 mg/dL even after a change in medication. We assessed the association between BTMs and the large decline in serum Ca levels using logistic regression analysis. ALP, BAP and TRACP-5b were significantly associated with a large decline in calcium after adjusting for age and sex (Table 4). During the 6-month period, one fracture occurred in the denosumab group (mandible = 1), while three occurred in the control group (fibula = 1, coccyx = 1, thoracic vertebra = 1).

**Table 4:** The effect of bone turnover markers on the high decline in serum calcium.

Variables	Univariate		Adjusted for age and sex	
	OR (95% CI)	p value	OR (95% CI)	p value
ALP, IU/L	1.015 (1.0007–1.039)	0.036	1.031 (1.005–1.076)	0.009
BAP, μg/L	1.202 (1.002–1.741)	0.044	1.747 (1.084–4.604)	0.007
TRACP-5b, mU/dL	1.004 (0.999–1.010)	0082	1.006 (1.000–1.015)	0.046

*OR* odds ratio, *CI* confidence interval.

## Discussion

In this retrospective non-controlled observational study, first, we showed that the patients treated with denosumab had increases in both lumbar spine BMD and total hip BMD at 6 months compared with control patients. Second, we showed that serum BTMs may reflect 6-month BMD increases in the lumbar spine and total hip compared with baseline. Third, we confirmed that the levels of BTMs were associated with a large decline in serum calcium levels after administrating denosumab, which was similar to the results of previous studies^14^.

In our study, many, but not all, patients treated with denosumab had BMD increases at 6 months. Considering this result, we analysed the factors that predict a better response to denosumab. One past study showed an association between pretreatment high BAP values and an increased effect on lumbar spine BMD during denosumab therapy among non-CKD women with osteoporosis^15^. Because denosumab decreased bone resorption and increased BMD by decreasing the number and activity of osteoclasts^5,6^, we hypothesized that high bone turnover status was associated with the increased effect of denosumab on BMD. The results obtained in this study were consistent with this hypothesis.

In our data, the denosumab group had significantly higher Adj Ca levels at baseline than the control group. This may be because we adjusted serum Adj Ca levels in the patients treated with denosumab to ≥ 9.0 mg/dL by increasing or adding an active vitamin D analogue or CaCO3 before initiating denosumab to prepare for the decline in serum Ca levels.

We showed that TRACP-5b, BAP and ALP were associated with a large decline in serum Adj Ca levels. The results were consistent with recent cohort studies of HD patients with osteoporosis treated with denosumab^13,14^.

In our investigation, high BTM levels were identified as a possible marker for predicting a good effect after initiating denosumab, and they were simultaneously shown to be a marker for a high risk of denosumab-associated hypocalcaemia, which is a well-known adverse effect. Patients with high bone turnover should be considered for initiation of treatment with denosumab because of the predicted benefit to BMD. However, such patients also need more careful monitoring of serum Ca levels to prevent a severe decline in calcium.

In our study, we monitored serum calcium three times a week for at least the first 2 weeks after denosumab initiation. If necessary, we increased supplementation with active vitamin D and calcium carbonate (CaCO3) or decreased calcimimetics, as appropriate. With careful monitoring of serum Ca, no cases of hypocalcaemia that required admission occurred.

There are some limitations of our study. First, this study was conducted at a single centre, and the study size was relatively small. A patient had a markedly high BMD change-rate in total hip, which possibly seemed to be caused by a measurement error. The small size of the cohort, including this outlier, may have influenced the outcome; therefore, our findings need to be confirmed in large sample studies. Second, the improvement of BMD could be influenced by the other medications—active vitamin D, CaCO3 and calcimimetics—and not by denosumab alone. However, active vitamin D, CaCO3 and calcimimetics were not associated with BMD changes in the simple regression analysis (Table 2). Third, before denosumab administration, three patients in the denosumab group had received treatment with bisphosphonates, which may have influenced the BTMs at baseline. Fourth, we did not conduct bone biopsies because they are invasive procedures and not routine management. Therefore, we could not evaluate the bone status, including oversuppression of bone turnover induced by denosumab.

In conclusion, we showed that high pretreatment bone turnover markers may be predictors of two conflicting events: good improvement of BMD and high risk of denosumab-induced hypocalcaemia. Among haemodialysis patients with high bone turnover status, denosumab administration with careful monitoring of calcium may be a useful option for the treatment of osteoporosis.

## Methods

### Study population

This 6-month observational cohort study was conducted at Masuko Memorial Hospital. The inclusion criteria were age ≥ 20 years, haemodialysis treatment for at least 3 months, and a diagnosis of osteoporosis in accordance with the Japanese Society for Bone and Mineral Research criteria: (1) patients having a prior fracture in either the lumbar spine or the proximal femur; (2) patients having a fragility fracture whose BMD was < 80% of Young Adult Mean (YAM); or (3) BMD was ≤ 70% or − 2.5 standard deviation (SD) of YAM^16^. The exclusion criteria included (1) unwillingness to provide consent for retrospective analysis of the data and (2) malignancy.

All patients who received denosumab provided written informed consent to be involved in the study. We recruited HD patients with osteoporosis who did not receive denosumab as control patients to compare the change in BMD. Participants were given written information about the research project and the opportunity to opt out. The study protocol was conducted in accordance with the Declaration of Helsinki. The hospital’s institutional review board approved this protocol (Ethics approval number: H25-40).

### Intervention

Eligible patients received a subcutaneous dose of 60 mg denosumab every 6 months. Because previous studies showed that denosumab leads to hypocalcaemia, especially during the first 2 weeks^17^, the patients received supplementation with a vitamin D analogue or CaCO3 to have serum albumin-adjusted Ca levels ≥ 9.0 mg/dL before initiation of treatment. We monitored serum Ca levels before every dialysis session during the first 2 weeks at least and modified the dosages of active vitamin D, calcium carbonate or calcimimetics, if necessary.

### Laboratory measurements

All patients treated with denosumab were scheduled dual X-ray absorptiometry (DXA) measurements of BMD at baseline and 6 months after treatment. Control patients underwent BMD measurement as a routine follow-up. The baseline of control patients was defined as the time of the first BMD measurement between June 2019 and July 2020. The BMD in the L2-L4 lumbar vertebrae and in the proximal total hip was measured using the Lunar iDXA system (GE Health Care Japan, Tokyo, Japan). Each percentage change in BMD at the lumbar spine and total hip from baseline to 6 months was calculated.

Baseline blood samples were drawn from the arteriovenous fistula just before an HD session. Serum levels of blood urea nitrogen (BUN), albumin-adjusted calcium (Adj. Ca), phosphate, magnesium, albumin, haemoglobin, ALP, and intact PTH were measured. Bone-specific alkaline phosphatase (BAP), tartrate-resistant acid phosphatase-5b (TRACP-5b), 1,25-dihydroxy vitamin D3 and 25 (OH) vitamin D were measured among the patients treated with denosumab at baseline. TRACP-5b was measured by enzyme immunoassays (SB bioscience, Tokyo, Japan) and BAP was measured by chemiluminescent enzyme immunoassay (Beckman Coulter Inc., Brea, CA, USA). If the serum albumin concentration was below 4.0 g/dL, Adj. Ca was calculated using Payne’s equation: Adj. Ca mg/dL = serum Ca mg/dL − serum albumin g/dL + 4.0.

### Outcomes

The primary outcome was the BMD changes in the lumbar spine and total hip 6 months after denosumab initiation. In our analyses, we included the data of the patients who underwent BMD measurements at both baseline and 6 months because the aim of the study was to identify the factors associated with BMD changes after treatment. The secondary outcome was adverse events.

### Adverse events

We monitored adverse events in patients treated with denosumab. We defined hypocalcaemia as an albumin-adjusted calcium (Adj Ca) level less than 8.0 g/dL. We also evaluated the Adj Ca decline at the nadir and defined a “large decline in serum calcium” as a maximum Adj Ca decline from baseline ≥ 1.0 mg/dL even after a change in medication to increase serum Ca levels or a ≥ 1.5 mg/dL decline in Adj Ca level without a change in medication, which was referenced from a previous study^13^.

### Statistical analyses

Continuous variables are expressed as the means ± SDs or medians with interquartile ranges (IQRs). For comparisons between two groups, Student’s t test or the Mann–Whitney U test was used for continuous variables, and the chi-square test was used for categorial variables. We used ANCOVA to compare the effect of denosumab on BMD between the denosumab and control groups and adjusted for confounding variables that were significantly different between the two groups. Simple and multiple regression analyses were performed to identify the independent factors for BMD increases. A logistic regression analysis was performed to assess the effects of variables on the large decline in serum Ca level. We considered p values < 0.05 to be statistically significant. All analyses were performed using JMP11 software (SAS Institute Inc., Cary, NC, USA).

### Ethical approval

This study was performed in accordance with the Helsinki Declaration and was approved by Masuko Memorial Hospital Ethics Committee (Ethics approval number: H25-40).

## Data Availability

The data collected for this study cannot be shared publicly because they contain information that could compromise the privacy of research participants. The data are available from the corresponding author upon request.
